# Altered Resting State Brain Dynamics in Temporal Lobe Epilepsy Can Be Observed in Spectral Power, Functional Connectivity and Graph Theory Metrics

**DOI:** 10.1371/journal.pone.0068609

**Published:** 2013-07-26

**Authors:** Maher A. Quraan, Cornelia McCormick, Melanie Cohn, Taufik A. Valiante, Mary Pat McAndrews

**Affiliations:** 1 Krembil Neuroscience Center and Toronto Western Research Institute, University Health Network, Toronto, Ontario, Canada; 2 Institute of Medical Sciences, University of Toronto, Toronto, Ontario, Canada; 3 Department of Psychology, University of Toronto, Toronto, Ontario, Canada; 4 Department of Neurosurgery, University of Toronto, Toronto, Ontario, Canada; Hangzhou Normal University, China

## Abstract

Despite a wealth of EEG epilepsy data that accumulated for over half a century, our ability to understand brain dynamics associated with epilepsy remains limited. Using EEG data from 15 controls and 9 left temporal lobe epilepsy (LTLE) patients, in this study we characterize how the dynamics of the healthy brain differ from the “dynamically balanced” state of the brain of epilepsy patients treated with anti-epileptic drugs in the context of resting state. We show that such differences can be observed in band power, synchronization and network measures, as well as deviations from the small world network (SWN) architecture of the healthy brain. The θ (4–7 Hz) and high α (10–13 Hz) bands showed the biggest deviations from healthy controls across various measures. In particular, patients demonstrated significantly higher power and synchronization than controls in the θ band, but lower synchronization and power in the high α band. Furthermore, differences between controls and patients in graph theory metrics revealed deviations from a SWN architecture. In the θ band epilepsy patients showed deviations toward an orderly network, while in the high α band they deviated toward a random network. These findings show that, despite the focal nature of LTLE, the epileptic brain differs in its global network characteristics from the healthy brain. To our knowledge, this is the only study to encompass power, connectivity and graph theory metrics to investigate the reorganization of resting state functional networks in LTLE patients.

## Introduction

Brain dynamics arise from complex interactions that depend on several factors including neuron type [1], [2], [3], synaptic properties [2], [3], gap junctions [4], [5] and synchronization of neural oscillations [6], [7], [8]. A delicate balance between these factors gives rise to a stable dynamical state [9] that characterizes the healthy brain. This stable dynamical state is characterized by a balance between excitation and inhibition [10]. When the network is dominated by excitation, a high neuronal firing occurs resulting in the impairment of neural processing [11]. In most models of epileptic seizures a transition from a balanced state to an excitatory dominated state results in a transition from the interictal to ictal state [12].

Epilepsy is of particular interest in understanding brain dynamics in general as the dynamical balance is only breached during a small time period over which a seizure occurs. This points to two important aspects that can shed some light on the functional impairment associated with epilepsy. Firstly, how do the dynamics of the healthy brain differ from the “dynamically balanced” state of the epileptic brain; and, secondly, how does the transition occur between this “dynamically balanced” state to an epileptic state [13], [14], [15], [16], [17], [18], [19], [20]? In this paper we pursue the first of these questions in the context of resting state dynamics.

While EEG and MEG data have been recorded for decades, renewed interest in resting state dynamics has recently emerged following numerous positron emission tomography (PET) and functional magnetic resonance imaging (fMRI) studies showing that spontaneous brain activity is not random. These studies revealed highly coherent functional networks closely related to the underlying anatomical structure [21]. While resting state networks (particularly the DMN network) have been well established in dozens of PET [22], [23] and fMRI [24], [25] studies for over a decade, electrophysiological data started emerging recently in healthy controls [26], [27], [28], [29] and epilepsy patients [30], [31], [32].

The realization that neurons constitute an electrophysiological dynamical system that is structurally and functionally connected has naturally led to the application of network theory to the study of brain dynamics. In the context of network theory, the physics of any complex system can be characterized by the same parameters despite profound differences in their constituent elements [33]. Network theory, also referred to as graph theory when displayed topologically as a set of nodes and edges [34], has shown great promise in understanding complex interactions in the healthy brain [35], as well as impaired network function resulting from various diseases [36], [37], [38]. A host of network metrics can be used to study network dynamics, many of which have been used in neuroimaging to characterize the healthy human brain [34], [35], [39], and to characterize differences associated with various neurologic and psychiatric disorders [40], [41], [42], [43], [44]. A main outcome of these studies is that interactions in the human brain possess small world network (SWN) properties that can be well distinguished from random networks and from regular networks. These properties have been attributed to evolutionary processes that attempt to balance cost and efficiency [34]. Several studies have reported deviations from SWN properties in various diseases [40], [43], [45], [46], [47].

Detailed network analyses from various structural and functional modalities have recently emerged in an attempt to provide a better understanding of network characteristics of the epileptic brain, changes in these characteristics over time and the relation of network measures to surgery outcome. In one study comparing cortical thickness connectivity in TLE patients and healthy controls, patients showed disruptions in various network measures, suggesting a reorganization of cortical thickness across brain networks. Longitudinal analysis demonstrated that network alterations intensify over time [48]. A resting state fMRI study comparing TLE patients and healthy controls showed deviations from the optimal SWN architecture [49] in patients. Deviations from SWN architecture in the θ band were reported to be related to greater vulnerability to seizures in an MEG study of individuals with tumor-related epilepsy [50]. An intracranial EEG study employed graph theory methods to identify critical network nodes in cortical networks during ictal and interictal states. A key network measure (betweenness centrality), was found to correlate with the location of the resected cortical regions in patients who were seizure free following surgical intervention [51]. In this regard, network measures provide a quantitative approach to characterizing complex network dynamics and can lead to a better understanding of epileptogenesis in addition to providing a valuable diagnostic and predictive tool [52], [53].

Despite a wealth of scalp EEG literature on epileptic seizures collectively pointing to an overall increase in synchronization during seizures [54], there are few studies that compare the resting state of healthy controls to that of epileptic patients in the interictal state at the network level. In one recent study [30] graph theory measures were applied to data from a 29-electrode EEG system. However, their study encompassed patients with different epileptic foci originating from one or both hemispheres, making it difficult to draw specific conclusions about the spatial extent and nature of the network disorder.

In this paper we use a 64-electrode EEG system to investigate various measures of brain dynamics at the local and network levels to characterize the properties of the epileptic brain in resting state and how it differs from the healthy brain. While it is well known that the dynamics of the epileptic brain differ from the healthy brain during seizures and transition to seizures, we show that differences at multiple levels can be observed during the “dynamically balanced” state at rest with no task demand. Our analysis extends across multiple frequency ranges, and examines band power, synchronization, and network measures for a more comprehensive evaluation of these differences in resting state dynamics.

## Materials and Methods

### Subjects

Fifteen healthy volunteers between the ages of 22 and 59 (mean age = 33±10) and nine individuals with left temporal lobe epilepsy (LTLE) between the ages 24 and 59 (mean age = 42±13) were recruited for this study. Healthy subjects were excluded if they had any neurological or psychiatric disorders or used psychotropic medication. LTLE patients were recruited from individuals who were determined to be candidates for resection surgery at Toronto Western Hospital and as such had focal seizures originating from the left medial temporal lobe that were refractory to medication. To determine seizure focus and surgical candidacy, patients were admitted to the Epilepsy Monitoring Unit and underwent continuous video-EEG monitoring concurrently with anti-epileptic drugs titration until a minimum of three seizures with unequivocal unilateral temporal onset were recorded. If independent bitemporal seizures were recorded, 80% of seizures were required to be on the same side. Most patients had left temporal lobe sclerosis as well as a history of febrile convulsions. Detailed patient data are provided in Table 1. All participants gave written consent to participate in this study which was approved by the hospital’s ethics committee.

**Table 1:** Clinical data for patients included in this analysis.

Subject ID	Age	Gender	Seizureduration	Age ofonset	AED	Dosage/day(mg)	Left medial temporal sclerosis	Febrile seizure
**207**	39	m	6	33	LTG TPM	400	yes	yes
						200		
**208**	24	m	4	20	LTG CLB	600	yes	unknown**
						30		
**220**	46	m	44	2	CBZ LTG	800	yes	yes
						300		
**301**	25	m	23	2	LEV SVAL	3000	yes	yes
						2000		
**302**	36	f	34	2	LEV TPM CBZ	1500	yes	Yes
						200		
						800		
**303**	59	m	58.5	0.5	CBZ LTG	600	yes	yes
						200		
**304**	59	m	11	48	CBZ TPM	800	no*	no
						300		
**305**	37	m	7	30	LTG CLB	400	yes	yes
						10		
**306**	54	f	27	27	CBZ LTG	1000	yes	yes
						500		

Antiepileptic drugs (AED): CBZ, Carbamazepine; CLB, Clobazam; LEV, Levetiracetam; LTG, Lamotrigine; TPM, Topiramate; SVAL, Sodium Valproate.

* Has sclerosis of the amygdale and hippocampus but not sufficient for an MTS diagnosis.

** Admitted to hospital for extended time as an infant, but exact circumstances are unknown.

### Data Acquisition

Subjects were asked to sit in a quiet room and to stay fully relaxed. A total of three minutes of EEG data were recorded with eyes closed (EC) and another three minutes with eyes open (EO). A Neuroscan 64-channel EEG system was used to record data at a sample rate of 500 Hz. Two (EOG) electrodes were used to record horizontal and vertical ocular artifacts.

While both the EC and EO condition have been used in various neuroimaging experiments to represent resting state, substantial differences are typically seen in scalp EEG between the two conditions. In particular, α power is typically substantially higher in most subjects in the EC than the EO condition and is known to contribute to brain connectivity. We thus chose to record data with both conditions.

### Data Analysis

#### Preprocessing

In the offline data preprocessing, each channel was re-referenced to the average of all channels to avoid systematic effects that may arise from referencing to a particular channel, particularly in the context of synchronization analysis [55], [56], [57]. A DC offset was subtracted based on the entire time range, and bad channels were removed then interpolated from neighboring channels.

Spectral power from EEG signals is generally difficult to quantify at low and high frequencies due to ocular and muscle artefacts in these regions. Signals from ocular artefacts are orders of magnitude higher than neural signals and result in a sharp rise in spectral power at low frequencies, while muscle artefacts make large contributions at high frequencies. Despite the various methods available to reduce such artefacts, determining their contribution to systematic errors is difficult to achieve in data with small statistics (with respect to both number of samples and number of subjects). The data was therefore further bandpassed in the 2 to 20 Hz range to avoid such artefacts. Remaining artefacts were removed manually by rejecting segments containing artefacts.

#### Spectral power

The cleaned data was fast Fourier transformed (FFT) to obtain the absolute spectral power for each channel in five frequency bands: δ (2–4 Hz), θ (4–7 Hz), α*_l_* (7–10 Hz), α*_h_* (10–13 Hz) and β (13–20 Hz). In order to allow power comparisons across bands of different width, the power spectrum was normalized by the frequency range resulting in units of µV/Hz.

We utilized the spectral power information to construct a single measure that can be used to quantify the observed differences across frequency bands between healthy controls and patients [58], [59], [60]. To this end, we used the power, *P,* in individual frequency bands to define a low-high asymmetry measure (*A_lh_*) such that.
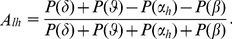
(1)


Similar measures have been previously constructed to characterize Alzheimer’s disease [58], [61] and depression [60] and, in the former, were correlated with clinical measures that characterize the severity of dementia. In epilepsy, an epileptogenicity index has been defined to quantify the degree of epileptogenicity using high frequency (rapid discharges) recorded with depth electrodes [62].

#### Functional connectivity

Synchronization of chaotic systems has captured tremendous interest in the field of non-linear dynamics and has been used in a wide range of scientific applications. Phase synchronization, a specific measure of synchronization which was first introduced by Rosenblum et al. (1996), found applications to time series recorded from brain activity soon after (Tass 1998, Mormann 2000). For a given signal *s(t)*, with a Hilbert transform *s’(t)*, the instantaneous phase difference *φ(t)* between two time series (labeled as *a* and *b*) is given by.
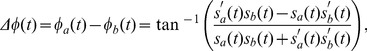
(2)and is confined to the interval [0,2π]. Following Mormann et al. (2000), we use a mean phase coherency measure of synchronization defined as
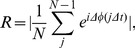
(3)where 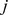 is the sample number and 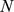 is the total number of samples. Mean phase coherency takes on values between 0 and 1, indicating no synchronization and full synchronization, respectively. In comparison to amplitude-based correlation measures, phase synchronization measures are less influenced by signal to noise fluctuations, and as such, result in a more robust measure of functional connectivity.

#### Network theory

Network theory can be represented geometrically as a set of nodes (representing processing centres) and edges that represent the information flow between them. This topological representation of the geometry is known as graph theory. In this context, a metric known as *path length* represents the number of edges traversed to go from one node to another. Overall, a network where a smaller number of edges are traversed on average to go from one node to another would be considered efficient. Hence, *efficiency* is defined as the inverse of *path length*. Another metric, *clustering coefficient*, indicates how connected a network is to its nearest neighbours. While random networks are characterized by high efficiency and low clustering coefficient, regular networks are characterized by low efficiency and high clustering coefficient. SWNs, on the other hand, lie somewhere in between, thus, balancing local specialization and global integration.

In the present analysis we used mean phase coherency described in the previous section to generate a connectivity matrix. The connectivity matrix was binarized at a preset proportional threshold to ensure that networks in the patients and control groups have the same number of edges so that group differences reﬂect alterations in network organization [48]. As there is no formal consensus on a robust method for threshold selection, based on previous studies [45], [49], we investigated a set of proportional thresholds over the range 0.3≤T≤0.6 corresponding to a degree range of 18.9≤K≤37.8 and a number of edges range of 1210≤D≤2420. A proportional threshold of 0.3 indicates that the strongest 30% of the connections were selected. The efficiency and clustering coefficient were calculated from the binary matrix using the Brain Connectivity Toolbox (BCT) [63].

For a network with *N* nodes belonging to set *G*, where the connectivity between any two nodes *(i,j)* is *a_ij_* ( = 1 if the connection exists and 0 otherwise), the clustering coefficient of node *i* is defined as [63], [64].
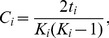
(4)where *K_i_* is the degree of node *i* defined as
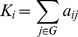
(5)and
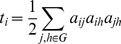
(6)is the number of triangles that can be formed between node 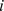 and its neighboring nodes.

The efficiency of the network is defined as.
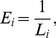
(7)where *L_i_* is the characteristic path length given by [63], [64]

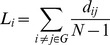
(8)and
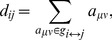
(9)where 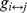 is the shortest path between nodes 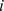 and 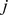. A network degree, absolute network clustering coefficient, and absolute network efficiency can be defined as
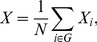
(10)where 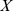 corresponds to 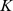, 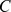 or 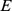, respectively

For a random network of N nodes and *K* network degree, the clustering coefficient of the network, *C_rand_* and characteristic path length *L_rand_* can be approximated as [45].
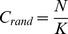
(11)and



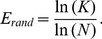
(12)In order to quantify SWN characteristics we computed a SWN parameter, σ, as [65].
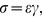
(13)where ε is the normalized efficiency of the network given by
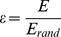
(14)and γ is the normalized clustering coefficient of the network given by


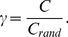
(15)

## Results

### Spectral Power

**Figure 1:**
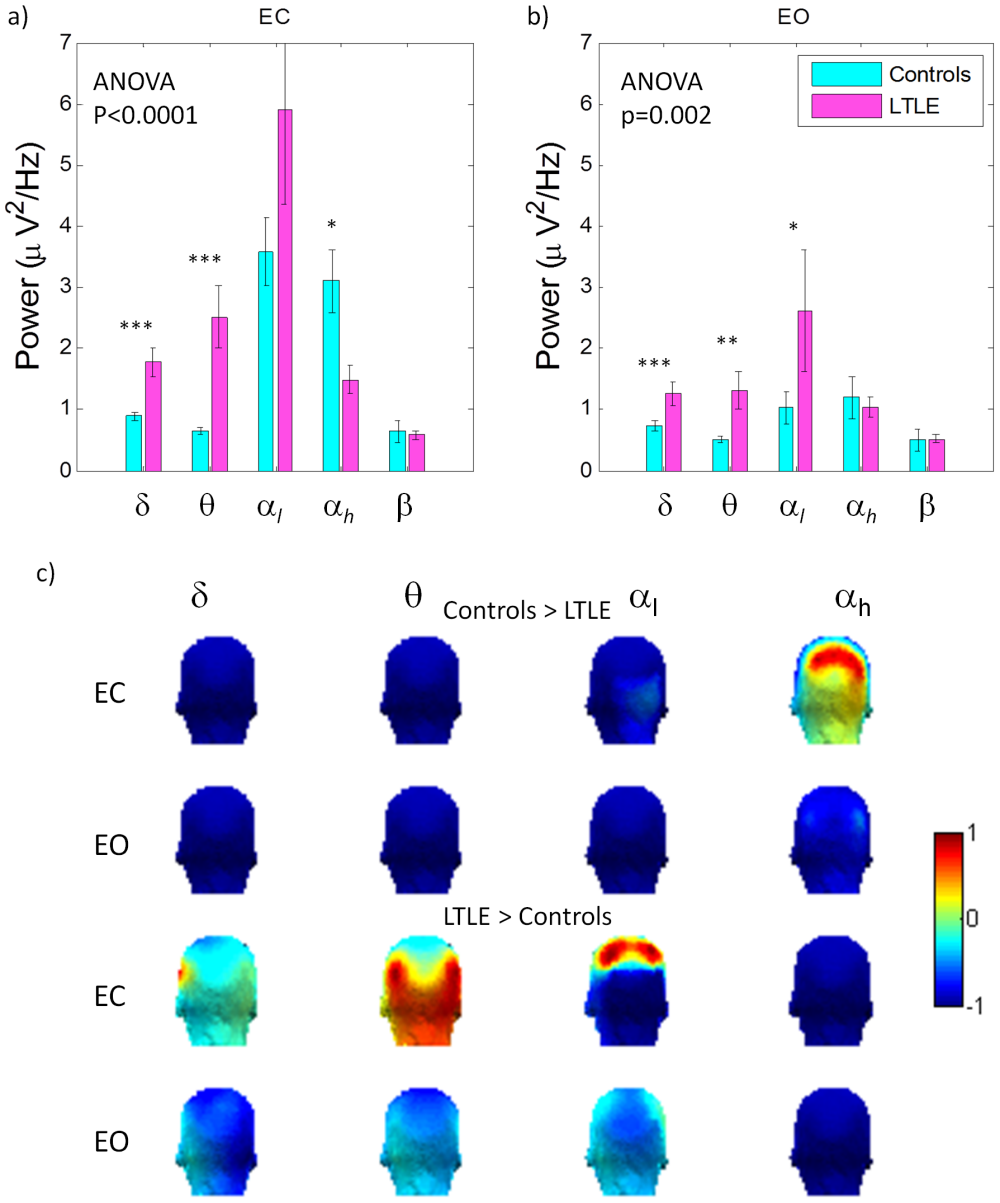
a) Total power over the range 2–20 Hz from healthy controls and LTLE patients for the EC and EO conditions. Figures b and c show the same across the various frequency bands as labeled on the figures for eyes closed and eyes open, respectively. Asterisks indicate where the difference between healthy controls and patients resulted in p<0.05 from a Kruskal-Wallis test. d) Topographical maps of spectral power for the EC and the EO condition showing the regions where power is greater in controls than patients (top) and where power is greater in patients than controls (bottom) for EC and EO as labelled.

Significant differences in band power between controls and patients were observed in the EC (ANOVA p<0.0001) and EO (ANOVA p = 0.002) conditions. Figure 1a shows power over the five frequency bands defined above. For the EC condition, an increase in power in patients relative to controls is observed in the δ (Kruskal-Wallis p<0.001) and θ (p<0.0004) bands. While the α*_l_* band shows higher power in patients than controls, the difference was not statistically significant due to large variations among subjects. In the α*_h_* band, on the other hand, patients show significantly lower spectral power than controls (Kruskal-Wallis p<0.03). For the EO condition (Figure 1b), statistically significant differences in spectral power were observed in the δ (Kruskal-Wallis p<0.001), θ (p<0.009) and α*_l_* (p<0.05) bands, with patients showing higher power than controls in all three bands. Table 2 summarizes these findings.

**Table 2:** Spectral power of healthy controls and LTLE patients for the eyes closed (EC) and eyes open (EO) conditions and the statistical significance of the difference as determined from a Kruskal-Wallis test.

Freq Band	Power (µV^2^/Hz)					
	EC			EO		
	HC	LTLE	p	HC	LTLE	p
***δ***	0.89	1.77	0.001	0.73	1.26	0.001
***θ***	0.65	2.52	0.0004	0.50	1.32	0.009
**α** ***_l_***	3.58	5.91	0.39	1.03	2.62	0.046
**α** ***_h_***	3.10	1.49	0.03	1.19	1.04	0.57
***β***	0.64	0.64	0.30	0.50	0.52	0.11

An investigation of the topography shows that the decrease in spectral power in LTLE patients in the α*_h_* band for the EC condition results mainly from highly focal activity in the parietal regions as shown in Figure 1c (controls>LTLE). The topographic maps are normalized to the maximum power in each power band for the purpose of showing the spatial distribution of power across the scalp for a given band across patients and controls as well as conditions. As such, a comparison across bands in Figure 1c would not be meaningful (this information can be obtained from Figures 1a and b). Figure 1c (LTLE>controls) shows that the increase in spectral power in the δ band results largely in an increase in power in the left temporal region for both the EC and the EO condition. In the θ band, the higher activity in patients with eyes closed results mainly from highly focal bilateral activity in the temporo-parietal regions. In the α*_l_* band with eyes closed, on the other hand, most of the increase is in the central channels.

Since our patients have left temporal lobe epilepsy we further investigated left-right power asymmetry in the two regions where most of the activity difference between controls and patients was observed, namely, the central and parietal-occipital channels. No power asymmetry was observed in these regions to within the statistical significance of our measurement in any of the five frequency bands, neither in controls nor patients (see Figure S3).

### Spectral Ratio Measures

**Figure 2:**
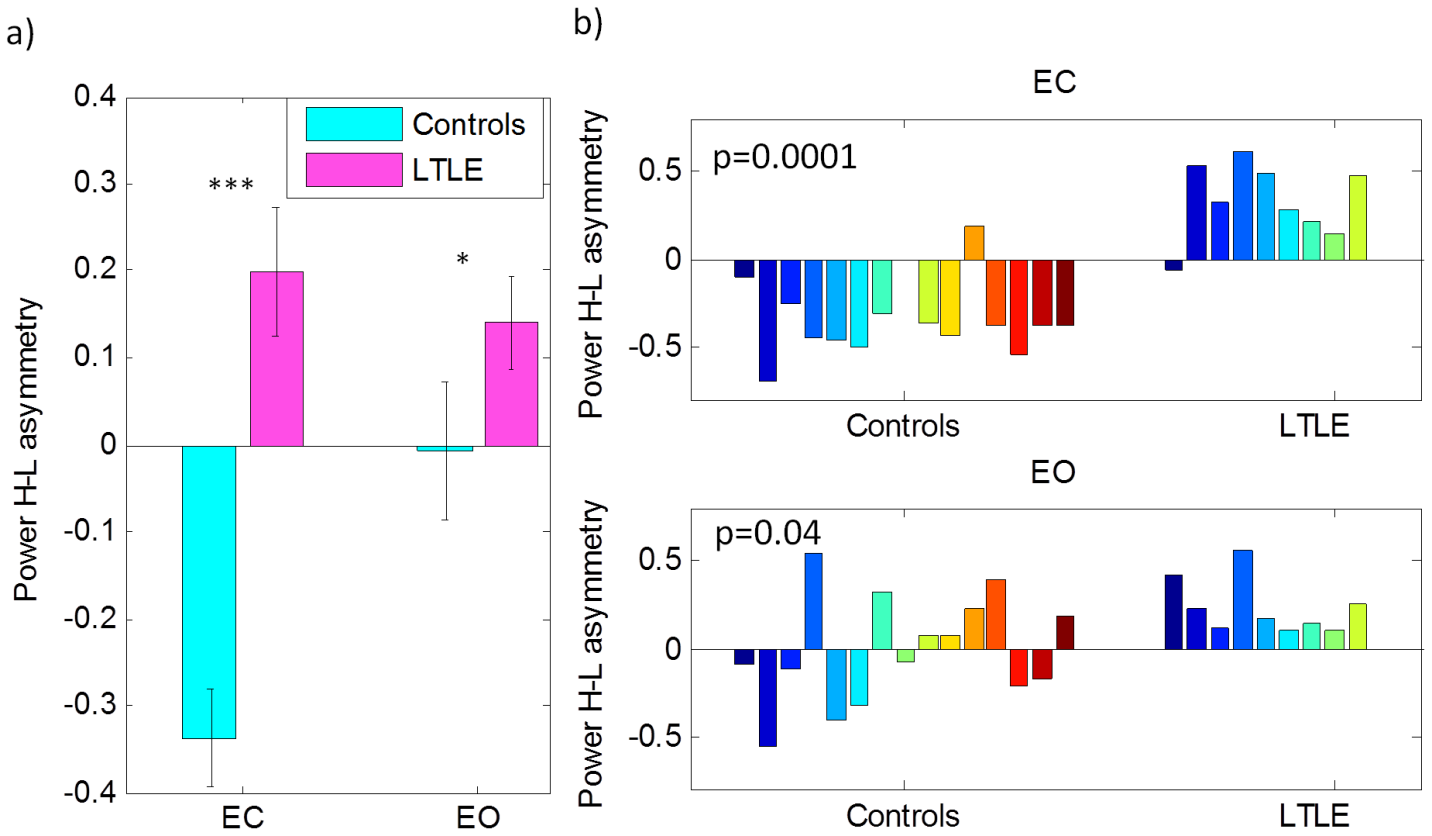
a) Group averaged power asymmetry (eqn. 1) contrasting brain activity in the high and low frequency bands, where a positive value indicates more power in the d and q bands compared to a*_h_* and b. The asterisks indicate statistical significance based on a Kruskal-Wallis test. The p vales are displayed on the plots in figure b. b) Same as figure a but for the individual subjects in the EC condition (top) and EO condition (bottom).

In order to utilize the observed differences in spectral power, we constructed a single power ratio measure that allowed us to distinguish patients from controls for the EC condition with very high accuracy, as shown in Figure 2a. This spectral power amounts to a low-high asymmetry (*A_lh_*) and can clearly distinguish between controls and patients in both the EC (Kruskal-Wallis p<0.0001) and the EO (p<0.04) conditions. Figure 2b shows the *A_lh_* for each individual subject. For the EC condition, the asymmetry results in *A_lh_*<0 for 14 out of 15 controls, while 8 out of 9 LTLE patients show *A_lh_* >0. For the EO condition, only 7 out of 15 controls showed *A_lh_* <0 while all patients showed *A_lh_* >0.

### Functional Connectivity

**Figure 3:**
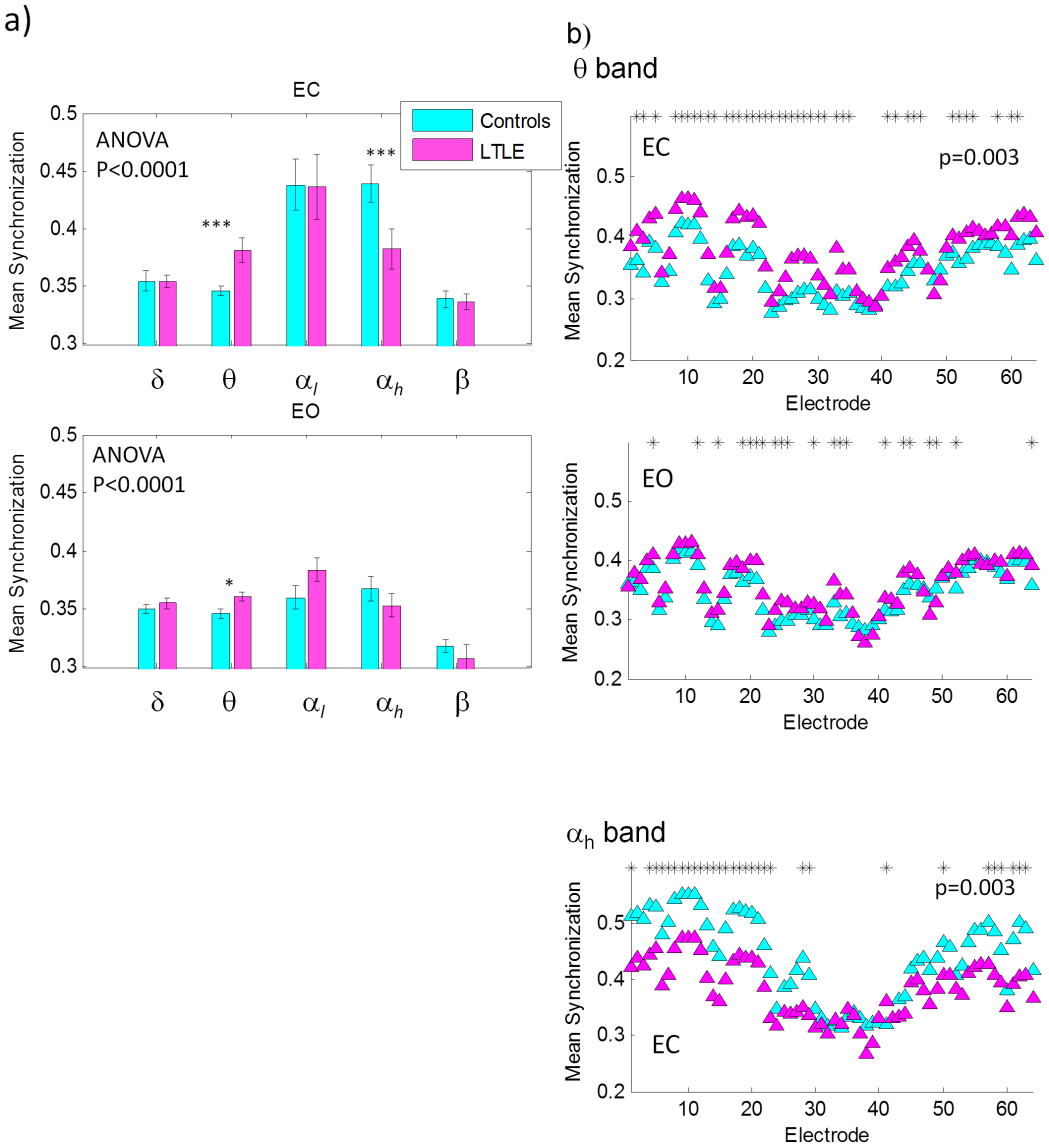
a) Mean synchronization computed by averaging synchronization between all pairs of electrodes in a given frequency band for EC (top) and EO (bottom). The asterisks indicate statistical significance in a Kruskal-Wallis test. b) Mean synchronization for every electrode computed by averaging the synchronization values between a given electrode and every other electrode in a given frequency band as labeled on the plots. The p values are obtained from a Kurkas-Wallis test and indicate statistical significance in the control to patient contrast for the specific band. Asterisks indicates channels that show statistically significant differences (p<0.05) between patients and controls.

**Figure 4:**
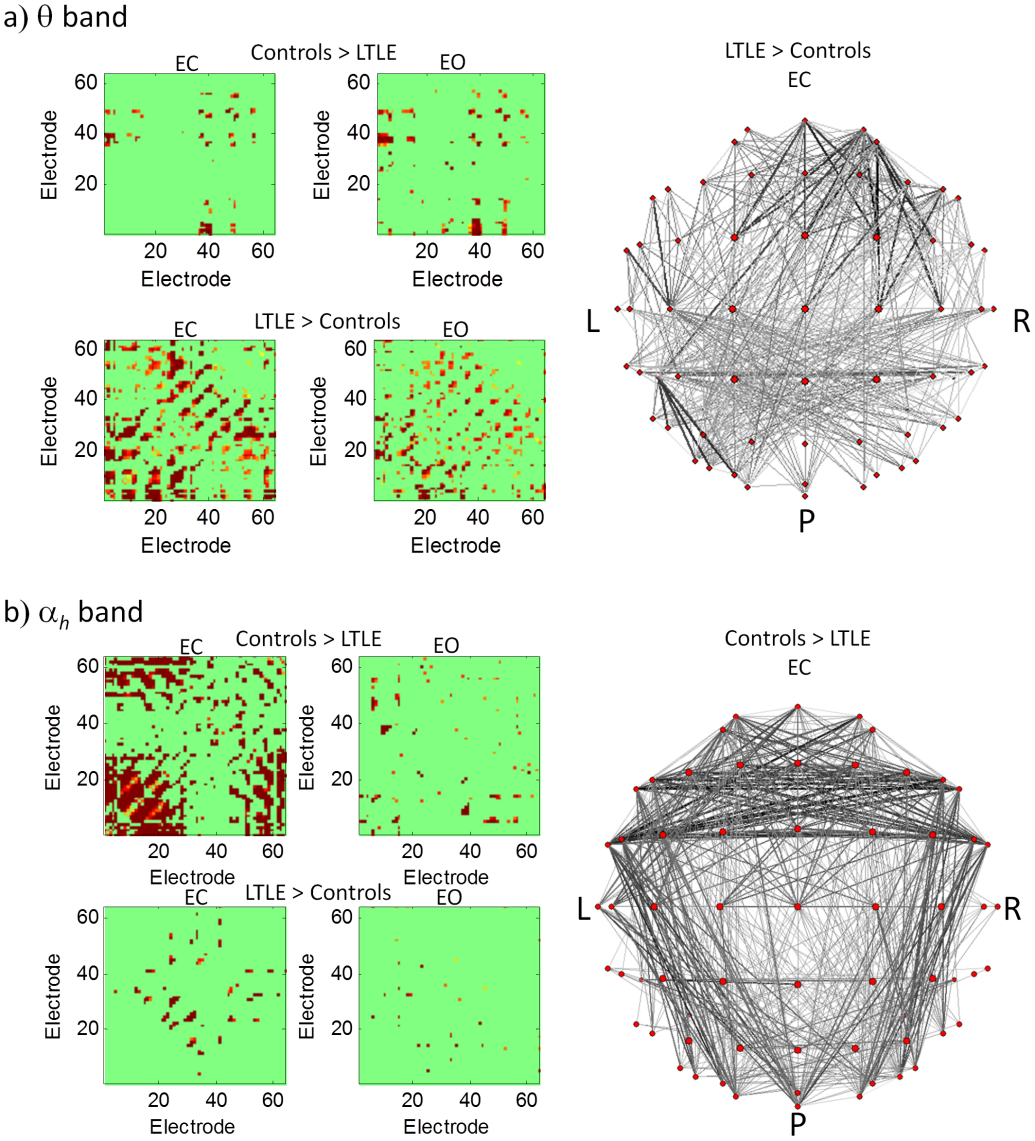
Connectivity matrices showing synchronization between each pair of electrodes in the a) q band and b) a*_h_* band for EC and EO as labeled on the plots. The spatial distribution is shown in the network diagrams in the right panels for the two cases where large differences were seen in the controls vs LTLE patient contrast. The line thickness indicates the connection strength. All figures are thresholded at p<0.05.

Significant differences in mean channel synchronization (calculated between a specific channel and all other channels then averaged) between controls and patients were observed in the EC (ANOVA p<0.0001) and EO (ANOVA p<0.0001) conditions across frequency bands. An investigation of the individual frequency bands showed significantly increased mean channel synchronization in the θ band in patients for both the EC (Kruskal-Wallis p = 0.003) and the EO (p = 0.02) conditions, but a significantly decreased synchronization in the α*_h_* band (p = 0.003) for EC as can be seen from Figure 3a. The synchronization of individual channels was also computed and plotted in Figure 3b in the bands that showed statistically significant differences between patients and controls to show how connectivity is distributed across channels (see Figure S1 for a channel map). The connectivity matrices in Figure 4 show the difference in synchronization between controls and patients for all pairs of electrodes and thus provide more detailed information on region to region interconnectivity. In the θ band (Figure 4a), higher synchronization in controls in the EC condition was confined to few electrode pairs showing mainly frontal-central connections. Increased connectivity in LTLE patients, on the other hand, was very wide spread and largely symmetric except for an increase in frontal-central right hemisphere connectivity. The EO condition revealed a similar pattern. In the α*_h_* band (Figure 4b), relative to LTLE patients controls showed a wide spread increased connectivity over the frontal and central regions (i.e. frontal-frontal, frontal-central and central-central), as well as increased connectivity in long range connections linking mainly occipital channels to frontal and central channels in the temporal regions.

### Graph Theory Metrics

**Figure 5:**
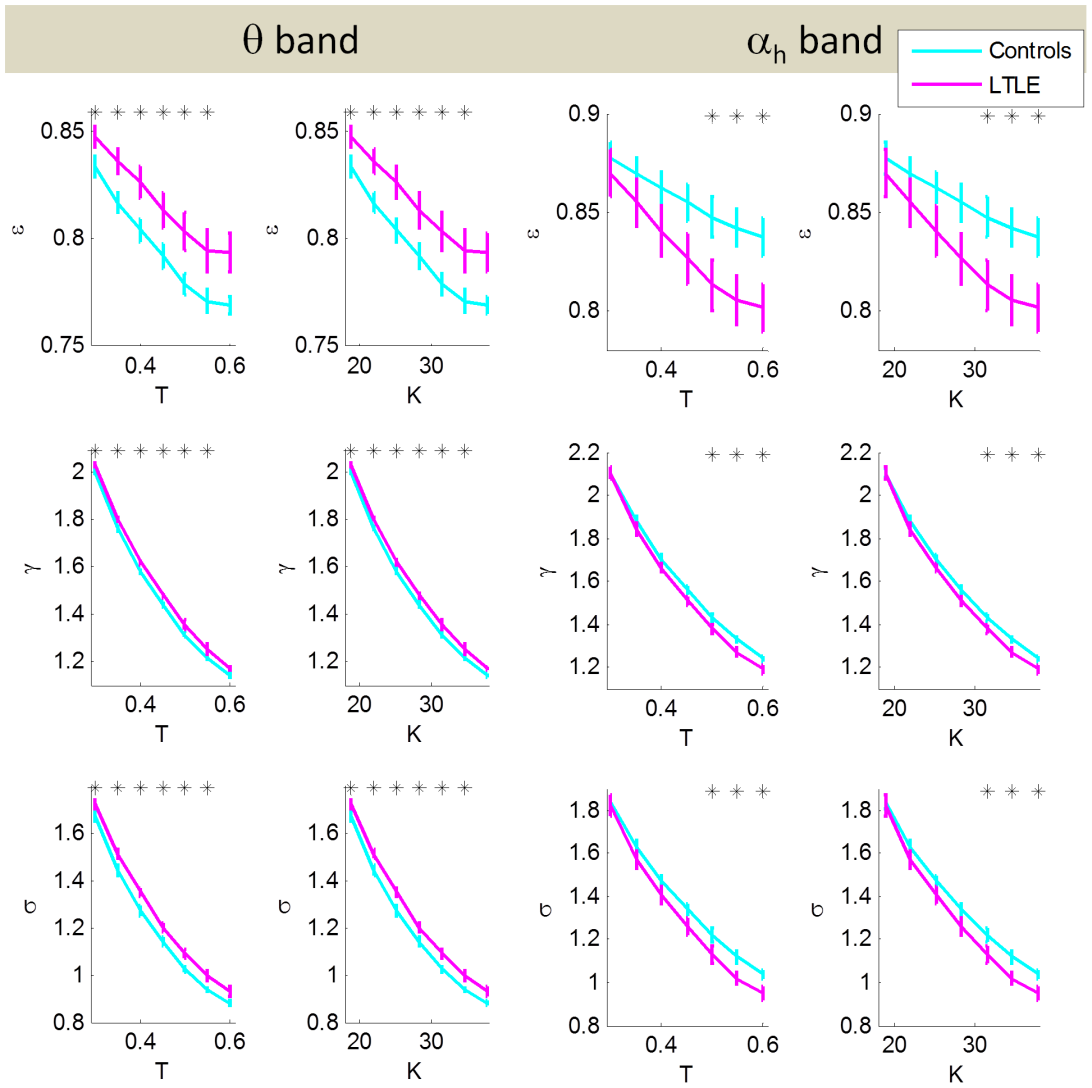
Graph theory metrics ε (top row), γ (middle row) and σ (bottom row) in the θ and α*_h_* bands as labelled on the figure. The metrics are plotted against a proportional threshold in the range 0.3<T<0.6 (first column) and the corresponding degree range of 18.9<K<37.8 (second column). Asterisks indicate statistical significance on a Kruskal-Wallis test.

**Figure 6:**
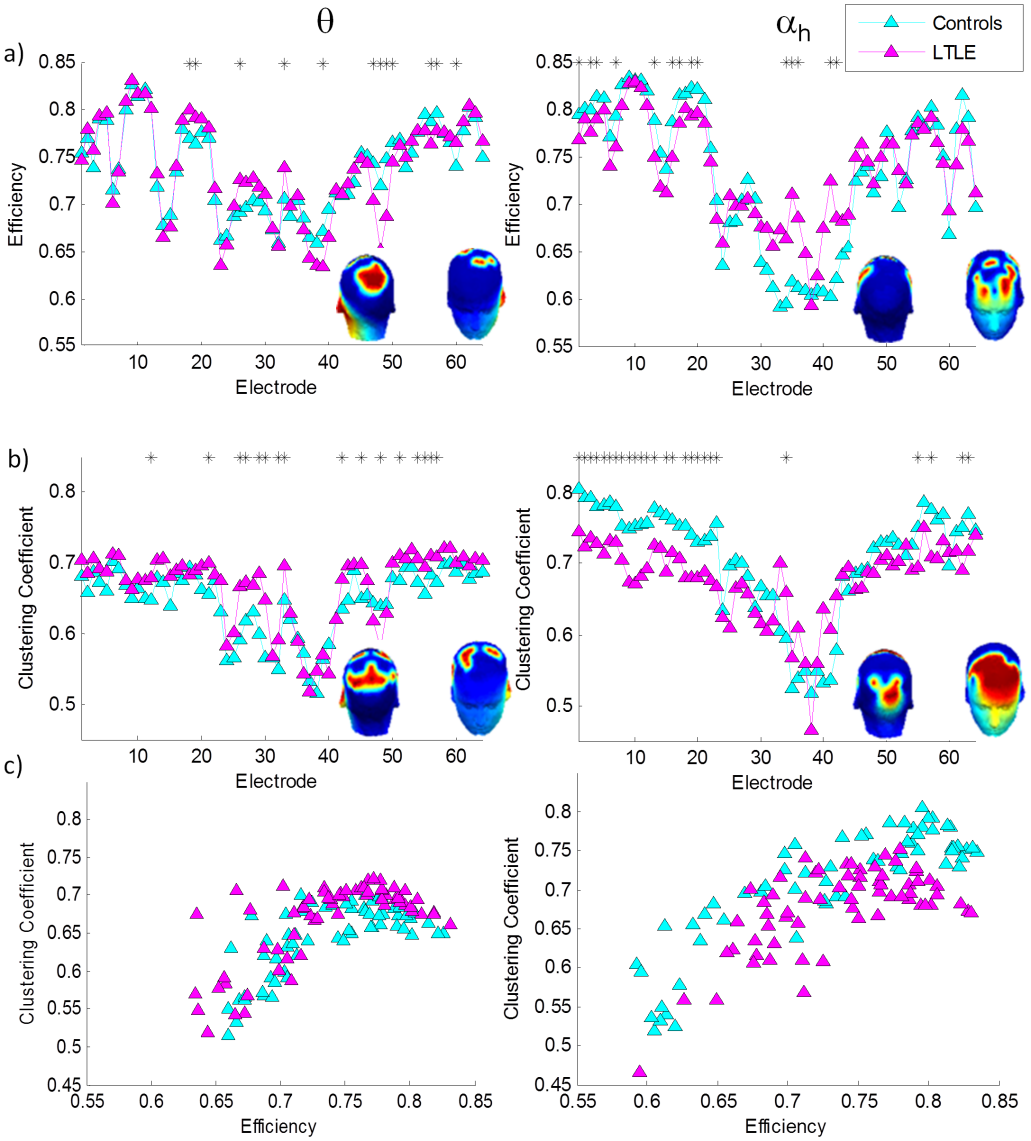
a) Graph theory efficiency metric for each electrode computed by averaging the efficiency of the electrode with all other electrodes. b) Graph theory clustering coefficient metric for each electrode. c) Clustering coefficient versus efficiency. For all three figures the left plots are for the q band while the right are for the a*_h_* band. The asterisks indicate statistical significance from a Kruskal-Wallis test. All results are for the EC condition. Topology of electrodes showing statistical significance on a Kurkas-Wallis test in figures a and b are shown in the abscissa.

We used graph theory metrics to gain further insight into the network properties of the epileptic brain and how it may deviate from a small world network (SWN). Since graph theory metrics are based on the functional connectivity results, only the θ and α*_h_* bands for the EC condition were investigated as they showed robust statistically significant differences between controls and patients (p<0.0001). Figure 5 shows normalized efficiency, ε; normalized clustering coefficient, γ; and small world index, σ, in the θ and α*_h_* bands as a function of a proportional threshold in the range 0.3<T<0.6 and as a function of the corresponding network degree. Following previous literature [49], [65], [66], we further investigated differences in network topology between the two groups and the relationship between efficiency and clustering coefficient at a sample proportional threshold of 0.5. At this threshold, differences between the patient and healthy controls are statistically significant in ε, γ and σ in the θ and α*_h_* bands. Figure 6 shows the results for EC in the two frequency bands that showed statistically significant synchronization differences between controls and patients. Electrodes that showed statistically significant differences between controls and patients in graph theory metrics are displayed topographically in each plot. In the θ band the channels showing higher efficiency in controls than patients are concentrated mainly in the mid parietal regions. The topography of channels that showed statistically significant differences in clustering coefficient are also highly concentrated in the parietal regions with patients showing higher clustering coefficient than patients. In order to gain further insight into deviations of these patterns from the SWN architecture, we plotted efficiency versus clustering coefficient in Figure 6c. As can be seen from this figure, patients showed a deviation from controls in their SWN architecture. As there is no formal procedure in the choice of graph theory thresholds the analysis results in Figure 6 are exploratory in nature.

## Discussion and Conclusions

In this paper, we investigated how spontaneous resting state activity from epilepsy patients differs from that of healthy controls. We showed that such differences can be observed using EEG at many levels encompassing spectral power, functional connectivity and graph theory measures and manifest as deviations from the optimal SWN architecture. Generally, various elements contribute to brain dynamics including neuron types, receptor bindings, gap junctions and connectivity. The ability to model how these components interact to give rise to a certain observable pattern is highly complex and beyond the scope of this work. Even in the context of epileptic seizures where the EEG recordings show substantial changes in the observed signals, the ability to fully identify the impairment in the underlying elements and their interactions is still lacking.

### Spectral Power

Investigation of band power revealed a very similar pattern in both EC and EO where the θ band showed higher power in patients than controls, while the α*_h_* band showed lower power in patients than controls. While the δ band also showed increased power in patients relative to controls in both the EC and the EO condition, the EO condition did not reach statistical significance. Spectral topography plots revealed that this increase results mainly from focal activity over the left temporal region. This is in agreement with previous studies that showed increased δ power on the side of the epileptogenic focus in TLE patients and was not changed by discontinuation of anti epileptic drugs (AEDs) [67]. An investigation of the topology in other frequency bands indicates wide spread differences in resting state dynamics between controls and patients that are largely left-right symmetric as seen in scalp EEG. This is not particularly surprising as EEG signals are dominated by cortical activations with little contributions from medial temporal lobe regions, and brain activity originating in deep structures unilaterally show little asymmetry in scalp electrical potential, making it difficult to detect an asymmetry with limited statistics. Furthermore, effects of AEDs would likely make a main contribution to this symmetric pattern. In particular, some AEDs have been shown to result in spectral slowing (i.e. a shift of the α peak to lower frequency, see Figure S2) [67], [68], but whether the observed effect can be entirely attributed to AEDs is difficult to assert from this data. One interesting feature is the wide spatial spread in spectral slowing where all electrodes showed a shift in the α peak toward lower frequency (Figure S2). However, in the EC condition, the temporal and central channels showed maximum shift exceeding 2 Hz. No asymmetry in spectral slowing was observed (Figure S3 shows the channels used to compute the asymmetry).

Alterations to the frequency spectra are well known in a variety of neural disorders. In particular, spectral slowing is known to occur in Parkinson disease related dementia [69], [70] and Alzheimer’s disease [59]. In Parkinson disease, it is generally believed that spectral slowing arises from a degeneration in the cholinergic system as treatment with the cholinterase inhibitor, revastigmine which breaks down acetylcholine, counteracts spectral slowing [69]. On the other hand, treatment with levedopa has shown no effect on spectral slowing arguing against the involvement of the dopaminergic system in the observed oscillatory dynamics [70].

While increases in low frequency power and decreases in high frequency power have been observed in Parkinson disease, Alzheimer’s disease and epilepsy, this is not the only direction in which impairments can manifest. In depression, a robust increase in high frequency oscillations has been observed that is highly consistent and reproducible across subjects [60].

We utilized differences in power across frequency bands to create a single measure, *A_lh_*. We showed that this low-high asymmetry spectral ratio can differentiate epilepsy patients from healthy controls with high accuracy. In the EC condition this measure resulted in 14 out of 15 controls having negative asymmetry scores, while 8 out of 9 patients had a positive asymmetry score. Such a measure would be particularly useful for the purpose of correlating brain-power impairments in epilepsy with clinical measures. The EO condition also showed a statistically significant difference in the group averaged asymmetry score, with all patients showing a positive score. However, scores from individual control subjects varied with only half the subjects showing negative scores.

### Functional Connectivity

Mounting evidence from empirical data recorded by various modalities and analyzed using various algorithms has accumulated showing that synchronization of neural activity is a manifestation of information exchange between different brain regions. Furthermore, it has been demonstrated that various diseases are associated with a change in synchronization from the dynamically balanced state recorded from the healthy brain. This impairment can result from a decrease in synchronization as is the case in Alzheimer’s disease [39], or an increase in synchronization resulting in an epileptic seizure [16]. Increased synchronization in epilepsy has been reported from scalp EEG and MEG recordings. Intracereberal recordings in TLE patients, in particular, have demonstrated that the increase during seizures results, at least partially, from an increase in synchronization between the thalamus and medial temporal lobe structures (hippocampus, entorhinal cortex and neocortex) [71]. While seizures have often been characterized as ‘hypersynchronous states’, several studies showed that this description is an oversimplification [72], [73]. In resting state, a complicated pattern emerged from this data. Synchronization differences between patients and controls were significant in the θ band for both EC and EO, where patients showed higher synchronization than controls. In the α*_h_* band, however, controls showed higher synchronization than patients in the EC condition, and the same was observed in the EO condition, although the difference did not reach statistical significance. This highlights two important aspects regarding the dynamics of the epileptic brain in resting state. Firstly, the disruption in synchronization does not only happen during epileptic seizures, but is in fact observable during resting state, and secondly, a complicated disruption in functional connectivity is observed in the resting state that does not amount to a simple increase (or decrease) in synchronization across all bands. In order to gain further insight into the characteristics of this disruption, we plotted the mean synchronization for each channel (Figure 3b) as well as the synchronization between all pairs of channels (Figure 4) for the bands that showed statistically significant differences between controls and patients. In the θ band, we found a wide spread increase in connectivity in patients relative to controls that was largely symmetric, although surprisingly, some frontal-central connections in the right hemisphere showed stronger connectivity. Whether this increase is indeed revealing a difference in LTLE patients relative to controls would be interesting to confirm with higher statistics. Moreover, connectivity from right temporal lobe epilepsy patients can shed some light on this finding.

In the α*_h_* band, controls showed higher synchronization than patients over a wide range, with a distinctly stronger connectivity across frontal and central regions. Moreover, stronger long range connectivity was found linking occipital regions with frontal and central regions in temporal areas in both hemispheres (see network diagrams Figure 4). Interestingly, connectivity across parietal channels showed the smallest difference, indicating that spectral slowing in parietal α is not likely to account for these findings.

Our result is in contrast to a recent study [30] that found increased broad band mean synchronization in epilepsy patients versus controls in resting state for both the EC (δ band was statistically significant) and EO (δ and β were significant) conditions. This study has also suffered from the same confound, as the majority of their patients were on two or more AEDs. Unlike our study, however, their patient population included those with epileptogenicity originating from either (or both) hemispheres and a range of focal origins. As the pathology and origin associated with each epilepsy type is different, it is vital to separate the different pathologies in order to uncover the specific disruptions in functional networks associated with each pathology. Additionally, our EEG system (64-channels) provided better spatial coverage than that of Horstmann et al. (29-channels). Yet, the differences between our results and those obtained by Horstmann et al. indicate that it is unlikely that the observed effect is entirely due to AEDs, as both patient groups where on similar AED combinations. The averaged functional connectivity across channels was not reported nor the spatial connectivity maps, both of which are likely to be of little utility considering the wide range of epileptogenicity considered in Horstmann et al.

Two independent studies on TLE patients using intracranial recordings [74] and fMRI [49], both of which provide high spatial specificity, have indicated increased synchronization in the medial temporal lobe regions. Another study combining fMRI and diffusion tensor imaging (DTI) found reduced structural and functional connectivity between the posterior cingulate cortex(PCC)/precuneus and the mTL regions in patients compared to healthy controls [75]. The comparison between these findings, however, is difficult to achieve considering the different modalities used, the different connectivity measures used, the different inclusion and exclusion criteria of patient groups and the different quantities reported (e.g. averaged connectivity in the mTL vs specific connections such as PCC-mTL). Collectively, however, they implicate functional connectivity in altered brain dynamics related to epilepsy.

### Graph Theory Metrics

Several studies have investigated changes in network parameters in different types of brain pathology. In schizophrenia [46], brain tumors [76], Alzheimer’s disease [39] and depression [66], a smaller clustering coefficient and a smaller path length (higher efficiency) compared to healthy controls were reported. In obsessive-compulsive disorder an increased clustering coefficient was reported [66]. In epilepsy, network analysis of cortical thickness correlations in a large cohort of TLE patients (N = 122) showed increased path length and clustering coefficient in patients relative to healthy controls suggesting a reorganization of structural networks. Furthermore, increased network disruption was associated with unfavorable postoperative seizure outcome indicating adverse effects to this network reorganization [48]. A resting state fMRI study revealed altered SWN properties in TLE patients compared to healthy controls, smaller clustering coefficient and shorter path length [49]. Another resting state fMRI study on idiopathic generalized epilepsy showed altered SWN architecture in patients compared to healthy controls [77]. An MEG study on tumor-related epilepsy, reported deviations from SWN architecture in the θ band [50].

In our study we found that the alteration in network measures depends on the frequency band considered. In the θ band, a higher clustering coefficient was found in patients relative to controls, while in the α*_h_* band, a lower clustering coefficient was found in patients relative to controls. This feature remained true for all proportional thresholds considered and was statistically significant over a wide threshold range (Figure 5). We further investigated the topology of these differences at a proportional threshold of 0.5. In the θ band, electrodes showing statistically significant differences between controls and patients were mainly in the parietal and central regions, while in the α*_h_* band they were widely distributed over the frontal region, with a more focused pattern in the occipital region. Since no formal procedure has been established in determining an appropriate graph theory thresholding scheme, we consider the analysis at this specific threshold to be exploratory in nature.

As the clustering coefficient signifies local processing, it is no surprise that we see reduced local processing in patients relative to controls in the θ band, as the θ rhythm is known to be associated with hippocampal function. Surprisingly, however, the biggest and most highly distributed reduction in local processing is seen in the frontal region and in the α*_h_* band, indicating that the pathology of this disease is by no means restricted to the medial temporal regions.

Since graph theory metrics summarize connectivity results using network-relevant measures, it is no surprise that our results would deviate from those of Horstmann et al. (2010) where the clustering coefficient and path length (inverse of efficiency) were both higher in patients than controls across all frequencies were the differences were statistically significant (clustering coefficient: δ and β in both EC and EO; path length: δ in EC and δ and θ in EO). No topology was reported in Horstmann et al. which is likely to be of little utility considering the diverse epiliptogeniety included in their analysis.

SWNs possess an optimal balance between modularized and distributed information processing. In order to examine the deviations of the epileptic brain in resting state from the SWN architecture of the healthy brain, we plotted the SWN index, σ, as a function of a preset proportional threshold (Figure 5). Deviations of patients from healthy controls were observed cross a wide range of thresholds. We further explored the relationship between efficiency and clustering coefficient at a sample threshold of 0.5. As it is already known that brain function reveals a SWN architecture [33], the distribution of our healthy controls represents such a distribution. A random network displayed in this fashion is characterized by high efficiency and low clustering coefficient and its nodes would therefore lie in the bottom right hand side of this graph. An orderly network is characterized by the opposite pattern and its nodes would lie in the upper left hand side (see Figure S2). In this context, in the θ band our epilepsy patients show a trend towards a random network, while in the α*_h_* band they show a trend toward an orderly network. This shows a different trend from most pathologies (see above) that typically indicate a trend toward randomness. During seizures, however, a trend toward an orderly network has been observed, characterized by an increase in clustering coefficient and increase in path length [73], [78]. Additionally, this result is consistent with findings in TLE [48], [49] other pathologies [66] in that the deviation from small worldlness is fairly small, indicating that the brain still follows the SWN architecture even in the diseased state and that small deviations in network parameters can lead to large behavioural impairments.

### Caveats and Future Directions

Scalp EEG data is inherently limited by poor spatial resolution making it difficult to relate the observed signals to the anatomical location from which they originate. Although various techniques have been used to localize sources from scalp EEG, such techniques remain poorly quantified particularly for deep sources. MEG provides higher spatial resolution as it is less susceptible to inhomogeneities in the brain and the poor conductivity of the skull. Furthermore, progress has been recently made in the detection and localization of deep sources in MEG using quantified analysis techniques [79], [80]. Despite this progress, many current methodologies are limited by various biases [81], [82], [83] making it difficult to evaluate functional connectivity in source space, particularly in the context of spatial filtering techniques such as beamformers, minimum norm and (s)LORETA [83], [84]. More promising techniques have been recently developed based on Baysian models [82], Kalman filters [85], [86] and particle filters [87]. While fMRI provides high spatial resolution, the slow hemodynamic response acts as a low pass filter obscuring the rich neural dynamics unfolding in the temporal domain. Furthermore, it remains unclear as to what physiological artifacts contribute to BOLD signals as the hemodynamic response is poorly understood. Simultaneous EEG and fMRI measurements provide another attempt at tackling this problem by combining fMRI’s high spatial resolution with EEG’s high temporal resolution [88], [89], [90]. Nonetheless, this approach also suffers from various technical limitations. Despite all these impediments, the knowledge currently accumulating from various modalities and methodologies, as well as the drive towards quantifying neuroscience, promises a better way forward in understanding the human brain in health and disease.

### Conclusions

Despite 80 years of EEG and over half a century of epilepsy recordings, our understanding of the dynamics underlying this most common neurological disorder is still limited. In this study we presented a characterization of brain dynamics accompanying the epileptic brain, encompassing brain activity, functional connectivity and network metrics in the hope of providing: 1) more stringent constraints on dynamical models as they must reproduce empirical data, and 2) providing quantified dynamical measures that can be correlated with clinical measures to shed some light on brain-behavior relationships. We showed that despite the fact that epileptic discharges are known to originate in the temporal lobe, the dysfunction is wide spread spatially and can be observed in spectral power, functional connectivity and graph theory metrics, further supporting evidence that epilepsy is a complex dynamical and structural disease [91]. Deviations in patients from controls in all these measures are frequency dependent. Higher power and higher connectivity is observed in the low frequency band (θ) in patients relative to controls, while the opposite is observed in the high frequency band (α*_h_*). While the low frequency band shows deviations in the epileptic brain toward an orderly network, the α*_h_* band reveals a deviation toward a random network. In agreement with previous measurements in other diseases the deviations from SWN was small highlighting the delicate balance required to achieve healthy brain dynamics. Due to the small sample size (patients, n = 9 and controls, n = 15), however, we emphasize that the generalization of our findings remains to be established by future work.

## Supporting Information

Figure S1 Channel positions of the Neuroscan system showing a front and a back view.
(TIF)

Figure S2 a) Spectral power from healthy controls and LTLE patients for the EC condition (top) and the EO condition (bottom) averaged over all electrodes. A spectral shift to the left in the patient group is observed in both conditions indicating spectral slowing. The p values displayed on the figures indicate the statistical significance of the α peak shift computed from a Kruskal-Wallis test. b) Topographical map of the spectral slowing (defined as the shift of the α peak) where zero indicates no slowing. The maximum slowing is seen in the temporal and central channels and exceeds 2 Hz.
(TIF)

Figure S3 Channels used in the calculation of the asymmetry below are enclosed in a box. The left central (LC) and right central (RC) channels were used to calculate the asymmetry, *A_C_*, in Figure S3 (top), while the left parieto-occipital (LPO) and right parieto-occipital (RPO) were used to calculate the asymmetry, *A_PO_*, in Figure S4 (bottom).
(TIF)

Figure S4 Left-right power asymmetry computed from the channels labeled in Figure S3.
(TIF)

## References

[1] Lawrence JJ, Saraga F, Churchill JF, Statland JM, Travis KE, et al (2006) Somatodendritic Kv7/KCNQ/M channels control interspike interval in hippocampal interneurons. J Neurosci 26: 12325–12338. 17122058 10.1523/JNEUROSCI.3521-06.2006 PMC6675427

[2] Cea-del Rio CA, Lawrence JJ, Tricoire L, Erdelyi F, Szabo G, et al (2010) M3 muscarinic acetylcholine receptor expression confers differential cholinergic modulation to neurochemically distinct hippocampal basket cell subtypes. J Neurosci 30: 6011–6024. 20427660 10.1523/JNEUROSCI.5040-09.2010 PMC2883452

[3] Lee CR, Tepper JM (2009) Basal ganglia control of substantia nigra dopaminergic neurons. J Neural Transm Suppl: 71–90. 10.1007/978-3-211-92660-4_6 20411769

[4] Munro E, Kopell N (2012) Subthreshold somatic voltage in neocortical pyramidal cells can control whether spikes propagate from the axonal plexus to axon terminals: a model study. J Neurophysiol 107: 2833–2852. 22378167 10.1152/jn.00709.2011

[5] Munro E, Borgers C (2010) Mechanisms of very fast oscillations in networks of axons coupled by gap junctions. J Comput Neurosci 28: 539–555. 20387109 10.1007/s10827-010-0235-6

[6] Contreras D, Steriade M (1997) Synchronization of low-frequency rhythms in corticothalamic networks. Neuroscience 76: 11–24. 8971755 10.1016/s0306-4522(96)00393-4

[7] Contreras D, Destexhe A, Sejnowski TJ, Steriade M (1996) Control of spatiotemporal coherence of a thalamic oscillation by corticothalamic feedback. Science 274: 771–774. 8864114 10.1126/science.274.5288.771

[8] Steriade M (1998) Corticothalamic networks, oscillations, and plasticity. Adv Neurol 77: 105–134. 9709820

[9] Deco G, Corbetta M (2011) The dynamical balance of the brain at rest. Neuroscientist 17: 107–123. 21196530 10.1177/1073858409354384 PMC4139497

[10] Coombes S, Terry JR (2012) The dynamics of neurological disease: integrating computational, experimental and clinical neuroscience. Eur J Neurosci 36: 2118–2120. 22805057 10.1111/j.1460-9568.2012.08185.x

[11] Ullah G, Cressman JR Jr, Barreto E, Schiff SJ (2009) The influence of sodium and potassium dynamics on excitability, seizures, and the stability of persistent states. J Comput Neurosci 26: 171–183. 19083088 10.1007/s10827-008-0130-6 PMC2951284

[12] Nymotin SA, Kerr CC, Francis JT, Lytton WW (2011) Training oscillatory dynamics with spike-timing-dependent plasticity in a computer model of neocortex; 2011 2011. IEEE.

[13] Zhang ZJ, Koifman J, Shin DS, Ye H, Florez CM, et al (2012) Transition to seizure: ictal discharge is preceded by exhausted presynaptic GABA release in the hippocampal CA3 region. J Neurosci 32: 2499–2512. 22396423 10.1523/JNEUROSCI.4247-11.2012 PMC6621818

[14] Zhang ZJ, Valiante TA, Carlen PL (2011) Transition to seizure: from “macro”- to “micro”-mysteries. Epilepsy Res 97: 290–299. 22075227 10.1016/j.eplepsyres.2011.09.025

[15] Singer BH, Derchansky M, Carlen PL, Zochowski M (2006) Lag synchrony measures dynamical processes underlying progression of seizure states. Phys Rev E Stat Nonlin Soft Matter Phys 73: 021910. 16605365 10.1103/PhysRevE.73.021910

[16] Mormann F, Andrzejak RG, Kreuz T, Rieke C, David P, et al (2003) Automated detection of a preseizure state based on a decrease in synchronization in intracranial electroencephalogram recordings from epilepsy patients. Phys Rev E Stat Nonlin Soft Matter Phys 67: 021912. 12636720 10.1103/PhysRevE.67.021912

[17] Wendling F, Chauvel P (2008) Transition To Ictal Activity In Temporal Lobe Epilepsy: Insights From Macroscopic Models. Computational Neuroscience in Epilepsy: 356–386.

[18] Chauviere L, Doublet T, Ghestem A, Siyoucef SS, Wendling F, et al (2012) Changes in interictal spike features precede the onset of temporal lobe epilepsy. Ann Neurol 71: 805–814. 22718546 10.1002/ana.23549

[19] Kramer MA, Eden UT, Kolaczyk ED, Zepeda R, Eskandar EN, et al (2010) Coalescence and fragmentation of cortical networks during focal seizures. J Neurosci 30: 10076–10085. 20668192 10.1523/JNEUROSCI.6309-09.2010 PMC2927849

[20] Kramer MA, Kolaczyk ED, Kirsch HE (2008) Emergent network topology at seizure onset in humans. Epilepsy Res 79: 173–186. 18359200 10.1016/j.eplepsyres.2008.02.002

[21] Deco G, Jirsa VK, McIntosh AR (2011) Emerging concepts for the dynamical organization of resting-state activity in the brain. Nat Rev Neurosci 12: 43–56. 21170073 10.1038/nrn2961

[22] Raichle ME, MacLeod AM, Snyder AZ, Powers WJ, Gusnard DA, et al (2001) A default mode of brain function. Proc Natl Acad Sci U S A 98: 676–682. 11209064 10.1073/pnas.98.2.676 PMC14647

[23] Geday J, Gjedde A (2009) Attention, emotion, and deactivation of default activity in inferior medial prefrontal cortex. Brain Cogn 69: 344–352. 18950928 10.1016/j.bandc.2008.08.009

[24] Damoiseaux JS, Rombouts SA, Barkhof F, Scheltens P, Stam CJ, et al (2006) Consistent resting-state networks across healthy subjects. Proc Natl Acad Sci U S A 103: 13848–13853. 16945915 10.1073/pnas.0601417103 PMC1564249

[25] Zhang S, Li CS (2012) Functional connectivity mapping of the human precuneus by resting state fMRI. Neuroimage 59: 3548–3562. 22116037 10.1016/j.neuroimage.2011.11.023 PMC3288461

[26] Chen AC, Feng W, Zhao H, Yin Y, Wang P (2008) EEG default mode network in the human brain: spectral regional field powers. Neuroimage 41: 561–574. 18403217 10.1016/j.neuroimage.2007.12.064

[27] Mantini D, Perrucci MG, Del Gratta C, Romani GL, Corbetta M (2007) Electrophysiological signatures of resting state networks in the human brain. Proc Natl Acad Sci U S A 104: 13170–13175. 17670949 10.1073/pnas.0700668104 PMC1941820

[28] Brookes MJ, Woolrich M, Luckhoo H, Price D, Hale JR, et al (2011) Investigating the electrophysiological basis of resting state networks using magnetoencephalography. Proc Natl Acad Sci U S A 108: 16783–16788. 21930901 10.1073/pnas.1112685108 PMC3189080

[29] de Pasquale F, Della Penna S, Snyder AZ, Lewis C, Mantini D, et al (2010) Temporal dynamics of spontaneous MEG activity in brain networks. Proc Natl Acad Sci U S A 107: 6040–6045. 20304792 10.1073/pnas.0913863107 PMC2851876

[30] Horstmann MT, Bialonski S, Noennig N, Mai H, Prusseit J, et al (2010) State dependent properties of epileptic brain networks: comparative graph-theoretical analyses of simultaneously recorded EEG and MEG. Clin Neurophysiol 121: 172–185. 20045375 10.1016/j.clinph.2009.10.013

[31] Chavez M, Valencia M, Navarro V, Latora V, Martinerie J (2010) Functional modularity of background activities in normal and epileptic brain networks. Phys Rev Lett 104: 118701. 20366507 10.1103/PhysRevLett.104.118701

[32] Douw L, de Groot M, van Dellen E, Heimans JJ, Ronner HE, et al (2010) ‘Functional connectivity’ is a sensitive predictor of epilepsy diagnosis after the first seizure. PLoS One 5: e10839. 20520774 10.1371/journal.pone.0010839 PMC2877105

[33] Strogatz SH (2001) Exploring complex networks. Nature 410: 268–276. 11258382 10.1038/35065725

[34] Bullmore E, Sporns O (2009) Complex brain networks: graph theoretical analysis of structural and functional systems. Nat Rev Neurosci 10: 186–198. 19190637 10.1038/nrn2575

[35] Bullmore ET, Bassett DS (2011) Brain graphs: graphical models of the human brain connectome. Annu Rev Clin Psychol 7: 113–140. 21128784 10.1146/annurev-clinpsy-040510-143934

[36] Meunier D, Achard S, Morcom A, Bullmore E (2009) Age-related changes in modular organization of human brain functional networks. Neuroimage 44: 715–723. 19027073 10.1016/j.neuroimage.2008.09.062

[37] Bassett DS, Bullmore E, Verchinski BA, Mattay VS, Weinberger DR, et al (2008) Hierarchical organization of human cortical networks in health and schizophrenia. J Neurosci 28: 9239–9248. 18784304 10.1523/JNEUROSCI.1929-08.2008 PMC2878961

[38] Alexander-Bloch AF, Gogtay N, Meunier D, Birn R, Clasen L, et al (2010) Disrupted modularity and local connectivity of brain functional networks in childhood-onset schizophrenia. Front Syst Neurosci 4: 147. 21031030 10.3389/fnsys.2010.00147 PMC2965020

[39] Stam CJ, de Haan W, Daffertshofer A, Jones BF, Manshanden I, et al (2009) Graph theoretical analysis of magnetoencephalographic functional connectivity in Alzheimer’s disease. Brain 132: 213–224. 18952674 10.1093/brain/awn262

[40] Jin SH, Lin P, Hallett M (2011) Abnormal reorganization of functional cortical small-world networks in focal hand dystonia. PLoS One 6: e28682. 22174867 10.1371/journal.pone.0028682 PMC3236757

[41] Ahmadlou M, Adeli H, Adeli A (2010) New diagnostic EEG markers of the Alzheimer’s disease using visibility graph. J Neural Transm 117: 1099–1109. 20714909 10.1007/s00702-010-0450-3

[42] Stam CJ, Reijneveld JC (2007) Graph theoretical analysis of complex networks in the brain. Nonlinear Biomed Phys 1: 3. 17908336 10.1186/1753-4631-1-3 PMC1976403

[43] de Haan W, Pijnenburg YA, Strijers RL, van der Made Y, van der Flier WM, et al (2009) Functional neural network analysis in frontotemporal dementia and Alzheimer’s disease using EEG and graph theory. BMC Neurosci 10: 101. 19698093 10.1186/1471-2202-10-101 PMC2736175

[44] Tahaei M, Jalili M, Knyazeva M (2012) Synchronizability of EEG-Based Functional Networks in Early Alzheimer’s Disease. IEEE Trans Neural Syst Rehabil Eng. 10.1109/TNSRE.2012.2202127 22695360

[45] Stam CJ, Jones BF, Nolte G, Breakspear M, Scheltens P (2007) Small-world networks and functional connectivity in Alzheimer’s disease. Cereb Cortex 17: 92–99. 16452642 10.1093/cercor/bhj127

[46] Micheloyannis S, Pachou E, Stam CJ, Breakspear M, Bitsios P, et al (2006) Small-world networks and disturbed functional connectivity in schizophrenia. Schizophr Res 87: 60–66. 16875801 10.1016/j.schres.2006.06.028

[47] Xie T, He Y (2011) Mapping the Alzheimer’s brain with connectomics. Front Psychiatry 2: 77. 22291664 10.3389/fpsyt.2011.00077 PMC3251821

[48] Bernhardt BC, Bernasconi N, Kim H, Bernasconi A (2012) Mapping thalamocortical network pathology in temporal lobe epilepsy. Neurology 78: 129–136. 22205759 10.1212/WNL.0b013e31823efd0d

[49] Liao W, Zhang Z, Pan Z, Mantini D, Ding J, et al (2010) Altered functional connectivity and small-world in mesial temporal lobe epilepsy. PLoS One 5: e8525. 20072616 10.1371/journal.pone.0008525 PMC2799523

[50] Douw L, van Dellen E, Baayen JC, Klein M, Velis DN, et al (2010) The lesioned brain: still a small-world? Front Hum Neurosci 4: 174. 21120140 10.3389/fnhum.2010.00174 PMC2991225

[51] Wilke C, Worrell G, He B (2011) Graph analysis of epileptogenic networks in human partial epilepsy. Epilepsia 52: 84–93. 21126244 10.1111/j.1528-1167.2010.02785.x PMC3200119

[52] Kramer MA, Cash SS (2012) Epilepsy as a disorder of cortical network organization. Neuroscientist 18: 360–372. 22235060 10.1177/1073858411422754 PMC3736575

[53] Richardson MP (2012) Large scale brain models of epilepsy: dynamics meets connectomics. J Neurol Neurosurg Psychiatry 83: 1238–1248. 22917671 10.1136/jnnp-2011-301944

[54] Lehnertz K, Bialonski S, Horstmann MT, Krug D, Rothkegel A, et al (2009) Synchronization phenomena in human epileptic brain networks. J Neurosci Methods 183: 42–48. 19481573 10.1016/j.jneumeth.2009.05.015

[55] Stam CJ, Nolte G, Daffertshofer A (2007) Phase lag index: assessment of functional connectivity from multi channel EEG and MEG with diminished bias from common sources. Hum Brain Mapp 28: 1178–1193. 17266107 10.1002/hbm.20346 PMC6871367

[56] Nunez PL, Srinivasan R, Westdorp AF, Wijesinghe RS, Tucker DM, et al (1997) EEG coherency. I: Statistics, reference electrode, volume conduction, Laplacians, cortical imaging, and interpretation at multiple scales. Electroencephalogr Clin Neurophysiol 103: 499–515. 9402881 10.1016/s0013-4694(97)00066-7

[57] Guevara R, Velazquez JL, Nenadovic V, Wennberg R, Senjanovic G, et al (2005) Phase synchronization measurements using electroencephalographic recordings: what can we really say about neuronal synchrony? Neuroinformatics 3: 301–314. 16284413 10.1385/NI:3:4:301

[58] Poza J, Hornero R, Escudero J, Fernandez A, Gomez C (2008) Analysis of spontaneous MEG activity in Alzheimer’s disease using time-frequency parameters. Conf Proc IEEE Eng Med Biol Soc 2008: 5712–5715. 19164014 10.1109/IEMBS.2008.4650511

[59] Poza J, Hornero R, Abasolo D, Fernandez A, Garcia M (2007) Extraction of spectral based measures from MEG background oscillations in Alzheimer’s disease. Med Eng Phys 29: 1073–1083. 17204443 10.1016/j.medengphy.2006.11.006

[60] Hinrikus H, Suhhova A, Bachmann M, Aadamsoo K, Vohma U, et al (2009) Electroencephalographic spectral asymmetry index for detection of depression. Med Biol Eng Comput 47: 1291–1299. 19911211 10.1007/s11517-009-0554-9

[61] Poza J, Hornero R, Abasolo D, Fernandez A, Escudero J (2007) Analysis of spontaneous MEG activity in patients with Alzheimer’s disease using spectral entropies. Conf Proc IEEE Eng Med Biol Soc 2007: 6180–6183. 18003432 10.1109/IEMBS.2007.4353766

[62] Bartolomei F, Chauvel P, Wendling F (2008) Epileptogenicity of brain structures in human temporal lobe epilepsy: a quantified study from intracerebral EEG. Brain 131: 1818–1830. 18556663 10.1093/brain/awn111

[63] Rubinov M, Sporns O (2010) Complex network measures of brain connectivity: uses and interpretations. Neuroimage 52: 1059–1069. 19819337 10.1016/j.neuroimage.2009.10.003

[64] Watts DJ, Strogatz SH (1998) Collective dynamics of ‘small-world’ networks. Nature 393: 440–442. 9623998 10.1038/30918

[65] Achard S, Salvador R, Whitcher B, Suckling J, Bullmore E (2006) A resilient, low-frequency, small-world human brain functional network with highly connected association cortical hubs. J Neurosci 26: 63–72. 16399673 10.1523/JNEUROSCI.3874-05.2006 PMC6674299

[66] Zhang J, Wang J, Wu Q, Kuang W, Huang X, et al (2011) Disrupted brain connectivity networks in drug-naive, first-episode major depressive disorder. Biol Psychiatry 70: 334–342. 21791259 10.1016/j.biopsych.2011.05.018

[67] Tuunainen A, Nousiainen U, Pilke A, Mervaala E, Partanen J, et al (1995) Spectral EEG during short-term discontinuation of antiepileptic medication in partial epilepsy. Epilepsia 36: 817–823. 7635101 10.1111/j.1528-1157.1995.tb01620.x

[68] Salinsky MC, Binder LM, Oken BS, Storzbach D, Aron CR, et al (2002) Effects of gabapentin and carbamazepine on the EEG and cognition in healthy volunteers. Epilepsia 43: 482–490. 12027908 10.1046/j.1528-1157.2002.22501.x

[69] Bosboom JL, Stoffers D, Wolters E, Stam CJ, Berendse HW (2009) MEG resting state functional connectivity in Parkinson’s disease related dementia. J Neural Transm 116: 193–202. 18982241 10.1007/s00702-008-0132-6

[70] Stoffers D, Bosboom JL, Wolters E, Stam CJ, Berendse HW (2008) Dopaminergic modulation of cortico-cortical functional connectivity in Parkinson’s disease: an MEG study. Exp Neurol 213: 191–195. 18590728 10.1016/j.expneurol.2008.05.021

[71] Guye M, Regis J, Tamura M, Wendling F, McGonigal A, et al (2006) The role of corticothalamic coupling in human temporal lobe epilepsy. Brain 129: 1917–1928. 16760199 10.1093/brain/awl151

[72] Li Y, Fleming IN, Colpan ME, Mogul DJ (2008) Neuronal desynchronization as a trigger for seizure generation. IEEE Trans Neural Syst Rehabil Eng 16: 62–73. 18303807 10.1109/TNSRE.2007.911084

[73] Ponten SC, Douw L, Bartolomei F, Reijneveld JC, Stam CJ (2009) Indications for network regularization during absence seizures: weighted and unweighted graph theoretical analyses. Exp Neurol 217: 197–204. 19232346 10.1016/j.expneurol.2009.02.001

[74] Bettus G, Wendling F, Guye M, Valton L, Regis J, et al (2008) Enhanced EEG functional connectivity in mesial temporal lobe epilepsy. Epilepsy Res 81: 58–68. 18547787 10.1016/j.eplepsyres.2008.04.020

[75] Liao W, Zhang Z, Pan Z, Mantini D, Ding J, et al (2011) Default mode network abnormalities in mesial temporal lobe epilepsy: a study combining fMRI and DTI. Hum Brain Mapp 32: 883–895. 20533558 10.1002/hbm.21076 PMC6870458

[76] Bartolomei F, Bosma I, Klein M, Baayen JC, Reijneveld JC, et al (2006) Disturbed functional connectivity in brain tumour patients: evaluation by graph analysis of synchronization matrices. Clin Neurophysiol 117: 2039–2049. 16859985 10.1016/j.clinph.2006.05.018

[77] Zhang Z, Liao W, Chen H, Mantini D, Ding JR, et al (2011) Altered functional-structural coupling of large-scale brain networks in idiopathic generalized epilepsy. Brain 134: 2912–2928. 21975588 10.1093/brain/awr223

[78] Ponten SC, Bartolomei F, Stam CJ (2007) Small-world networks and epilepsy: graph theoretical analysis of intracerebrally recorded mesial temporal lobe seizures. Clin Neurophysiol 118: 918–927. 17314065 10.1016/j.clinph.2006.12.002

[79] Quraan MA, Moses SN, Hung Y, Mills T, Taylor MJ (2011) Detection and localization of hippocampal activity using beamformers with MEG: a detailed investigation using simulations and empirical data. Hum Brain Mapp 32: 812–827. 21484951 10.1002/hbm.21068 PMC6870394

[80] Mills T, Lalancette M, Moses SN, Taylor MJ, Quraan MA (2012) Techniques for Detection and Localization of Weak Hippocampal and Medial Frontal Sources Using Beamformers in MEG. Brain Topogr 25: 248–263. 22350670 10.1007/s10548-012-0217-2

[81] Quraan MA, Cheyne D (2010) Reconstruction of correlated brain activity with adaptive spatial filters in MEG. Neuroimage 49: 2387–2400. 19850135 10.1016/j.neuroimage.2009.10.012

[82] Wipf DP, Owen JP, Attias HT, Sekihara K, Nagarajan SS (2010) Robust Bayesian estimation of the location, orientation, and time course of multiple correlated neural sources using MEG. Neuroimage 49: 641–655. 19596072 10.1016/j.neuroimage.2009.06.083 PMC4083006

[83] Quraan MA (2011) Characterization of brain dynamics using beamformer techniques: advantages and limitations. In: Pang EW, editor. Magnetoencephelaography. Croatia: INTECH. 67–92.

[84] Schoffelen JM, Gross J (2009) Source connectivity analysis with MEG and EEG. Hum Brain Mapp 30: 1857–1865. 19235884 10.1002/hbm.20745 PMC6870611

[85] Long CJ, Purdon PL, Temereanca S, Desai NU, Hamalainen MS, et al (2011) State-Space Solutions to the Dynamic Magnetoencephalography Inverse Problem Using High Performance Computing. Ann Appl Stat 5: 1207–1228. 22081780 10.1214/11-AOAS483 PMC3212953

[86] Long CJ, Purdon RL, Temereanca S, Desai NU, Hamalainen M, et al (2006) Large scale Kalman filtering solutions to the electrophysiological source localization problem–a MEG case study. Conf Proc IEEE Eng Med Biol Soc 1: 4532–4535. 17947095 10.1109/IEMBS.2006.259537

[87] Campi C, Pascarella A, Sorrentino A, Piana M (2011) Highly Automated Dipole EStimation (HADES). Comput Intell Neurosci 2011: 982185. 21437232 10.1155/2011/982185 PMC3061326

[88] Meyer MC, van Oort ES, Barth M (2012) Electrophysiological Correlation Patterns of Resting State Networks in Single Subjects: A Combined EEG-fMRI Study. Brain Topogr. 10.1007/s10548-012-0235-0 PMC3536973 22752947

[89] Murta T, Leal A, Garrido MI, Figueiredo P (2012) Dynamic Causal Modelling of epileptic seizure propagation pathways: A combined EEG-fMRI study. Neuroimage. 10.1016/j.neuroimage.2012.05.053 PMC3778869 22634857

[90] Pouliot P, Tremblay J, Robert M, Vannasing P, Lepore F, et al (2012) Nonlinear hemodynamic responses in human epilepsy: a multimodal analysis with fNIRS-EEG and fMRI-EEG. J Neurosci Methods 204: 326–340. 22138633 10.1016/j.jneumeth.2011.11.016

[91] Bell B, Lin JJ, Seidenberg M, Hermann B (2011) The neurobiology of cognitive disorders in temporal lobe epilepsy. Nat Rev Neurol 7: 154–164. 21304484 10.1038/nrneurol.2011.3 PMC3856217

