# Supplementary figures and images for: Altered Resting State Brain Dynamics in Temporal Lobe Epilepsy Can Be Observed in Spectral Power, Functional Connectivity and Graph Theory Metrics
Source: PLoS One. 2013 Jul 26;8(7):e68609. doi: 10.1371/journal.pone.0068609 (PMC3724835; doi:10.1371/journal.pone.0068609)

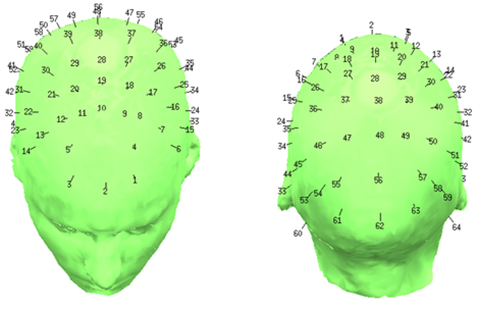

Supplement: Figure S1 — Channel positions of the Neuroscan system showing a front and a back view. (TIF) [file pone.0068609.s001.tif]

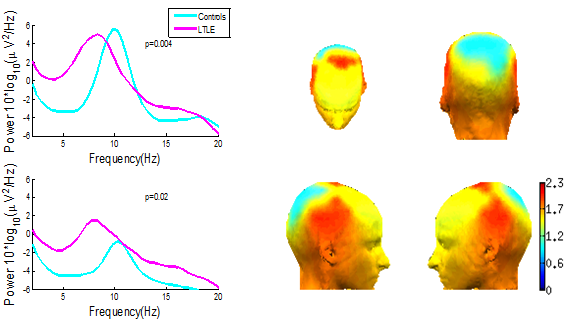

Supplement: Figure S2 — a) Spectral power from healthy controls and LTLE patients for the EC condition (top) and the EO condition (bottom) averaged over all electrodes. A spectral shift to the left in the patient group is observed in both conditions indicating spectral slowing. The p values displayed on the figures indicate the statistical significance of the α peak shift computed from a Kruskal-Wallis test. b) Topographical map of the spectral slowing (defined as the shift of the α peak) where zero indicates no slowing. The maximum slowing is seen in the temporal and central channels and exceeds 2 Hz. (TIF) [file pone.0068609.s002.tif]

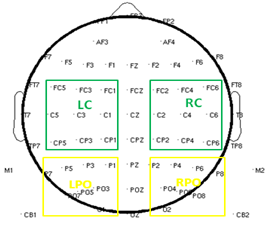

Supplement: Figure S3 — Channels used in the calculation of the asymmetry below are enclosed in a box. The left central (LC) and right central (RC) channels were used to calculate the asymmetry, AC, in Figure S3 (top), while the left parieto-occipital (LPO) and right parieto-occipital (RPO) were used to calculate the asymmetry, APO, in Figure S4 (bottom). (TIF) [file pone.0068609.s003.tif]

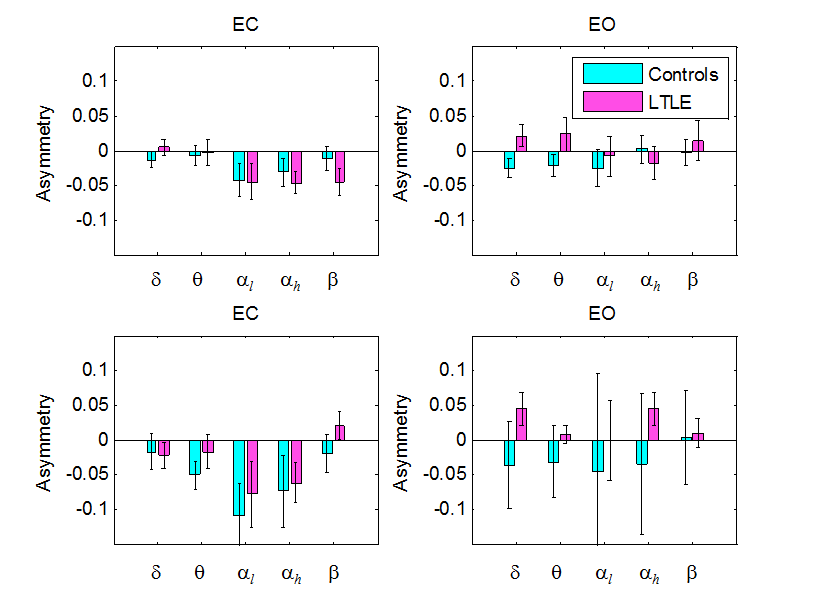

Supplement: Figure S4 — Left-right power asymmetry computed from the channels labeled in Figure S3. (TIF) [file pone.0068609.s004.tif]
